# The feasibility of following up prisoners, with mental health problems, after release: a pilot trial employing an innovative system, for engagement and retention in research, with a harder-to-engage population

**DOI:** 10.1186/s13063-018-2911-1

**Published:** 2018-10-01

**Authors:** Cath Quinn, Richard Byng, Deborah Shenton, Cordet Smart, Susan Michie, Amy Stewart, Rod Taylor, Mike Maguire, Tirril Harris, Jenny Shaw

**Affiliations:** 1 Plymouth University Peninsula Schools of Medicine and Dentistry, Drake Circus, Plymouth, Devon PL4 8AA UK; 2 Plymouth University, Drake Circus, Plymouth, Devon PL4 8AA UK; 3 University College London, Gower Street, London, WC1E 6BT UK; 4 University of Exeter Medical School, St Luke’s Campus, Exeter, EX1 2LU UK; 5 University of South Wales, Pontypridd, CF37 1DL UK; 6 King’s College London, Strand, London, WC2R 2LS UK; 7 The University of Manchester, Oxford Rd, Manchester, M13 9PL UK

**Keywords:** Prisoner, Offender, Mental health, Randomised controlled trial, Follow-up procedures

## Abstract

**Background:**

Following up released prisoners is demanding, particularly for those prisoners with mental health problems, for whom stigma and chaotic lifestyles are problematic. Measurement of mental health outcomes after release is challenging. To evaluate mental healthcare for offender populations, using high-quality randomised controlled trials, evidenced-based methods must be developed to engage them while in custody, to locate and re-interview them after release, and to collect potentially stigmatising mental health outcomes data.

**Methods:**

We developed an initial theoretical model and operational procedures for collecting baseline and follow-up data informed by a literature search, focus groups, and case studies. Male prisoners from five prisons in two sites were invited to participate. The inclusion criteria included individuals who were above threshold on nine-item Patient Health Questionnaire, seven-item Generalized Anxiety Disorder, or post-traumatic stress disorder scales, or who had reported mental health problems in the past 2 years or had been assessed with a likely personality disorder. Potential participants were interviewed to generate baseline data and were re-contacted before their release. We then contacted them for a follow-up interview, which included repeating the earlier data collection measures 2–8 weeks after release. A qualitative formative process evaluation produced and refined a model procedure for the recruitment and retention of male prison leavers in trials, identified the mechanisms which promoted engagement and retention, and mapped these against a theoretical behaviour change model.

**Results:**

We developed a flexible procedure which was successful in recruiting male prison leavers to a pilot trial: 185/243 (76%, 95% confidence interval (CI) 70–81%) of those approached agreed to participate. We also retained 63% (95% CI 54–71%) of those eligible to participate in a follow-up interview 2–8 weeks after release. Mental health outcomes data was collected at both these time points.

**Conclusions:**

It is possible to design acceptable procedures to achieve sustained engagement critical for delivering and evaluating interventions in prison and in the community and to collect mental health outcomes data. These procedures may reduce attrition bias in future randomised controlled trials of mental health interventions for prison leavers. This procedure has been replicated and successfully delivered in a subsequent pilot trial and a definitive randomised controlled trial.

**Electronic supplementary material:**

The online version of this article (10.1186/s13063-018-2911-1) contains supplementary material, which is available to authorized users.

## Background

Prisoners have high levels of common mental health problems (anxiety and depression), with comorbid substance misuse and personality dysfunction being frequently reported [1]. The provision of and access to mental health interventions for common mental health problems for prisoners and for offenders in the community are poor, with healthcare systems and offenders themselves both contributing to low levels of access to routine care [2]. Therapeutic and organisational interventions for prison leavers with anxiety and depression have not been evaluated in randomised controlled trials.

Healthcare service’s engagement with offenders is often problematic; offenders often distrust healthcare professionals and do not want to perceive themselves as having potentially stigmatising mental health problems [3]. Housing, relationships, and employment are often higher priorities for prisoners on their release than accessing health services [4]. Development of evidence-based interventions for engaging and retaining offenders, and prison leavers in particular, is therefore a priority for services; we suggest that it is also critical for the conduct of successful trials.

The development of clinical and service interventions that aim to change behaviours has been the subject of considerable research for the wider population and draws on a large and complex area of social influence and change. Theoretical approaches to understanding motivation and behaviour change are diverse, with a range of models developed. The Behaviour Change Wheel has been synthesised from 19 frameworks of behaviour change with a model of behaviour, the ‘COM-B system’, as the hub. The COM-B system posits that three conditions — capability, opportunity, and motivation — are essential for behaviour change. These conditions are linked to nine intervention functions to consider using when designing protocols to improve the likelihood of successfully achieving change [5]. This expanding field of health services research can be drawn on to develop models of care and research procedures for prison leavers.

Sustained contact with the Criminal Justice System, through community or custodial supervision, is a key opportunity to deliver interventions to offenders and evaluate them in randomised controlled trials [2]. Prisoners are in a fixed location, are relatively compliant, are disconnected from their usual social context, and are often bored, which means that initial recruitment to studies can be high [6, 7]. However, sustained engagement on release, and therefore achievement of adequate follow-up rates, has been problematic for both descriptive studies [8] and trials of both health and criminal justice interventions [9–11]. The main exception is for prisoners receiving interventions that they particularly value, such as opiate substitution for substance misuse (65% and 99% follow-up rates) [12, 13]. The highest follow-up rates achieved in other groups include prisoners with HIV receiving antiretroviral medication (50% and 72% in the USA) [14, 15] and female offenders receiving very specific interventions, for example, help with breastfeeding infants (85% 12 months after release and 59% 3 years after release) [16]. No prison trials of interventions for individuals with common mental health problems have been identified.

The challenges of engaging with and following up offenders must be addressed, particularly for those serving numerous short-term sentences, who are less likely to have received pre-release support, so that healthcare and treatment can be evaluated in high-quality randomised controlled trials and delivered to offender populations. We undertook a study to develop and then test a feasible and acceptable procedure to achieve the sustained engagement critical for delivering and evaluating interventions both in prison and in the community after release.

## Methods

**Fig. 1:**
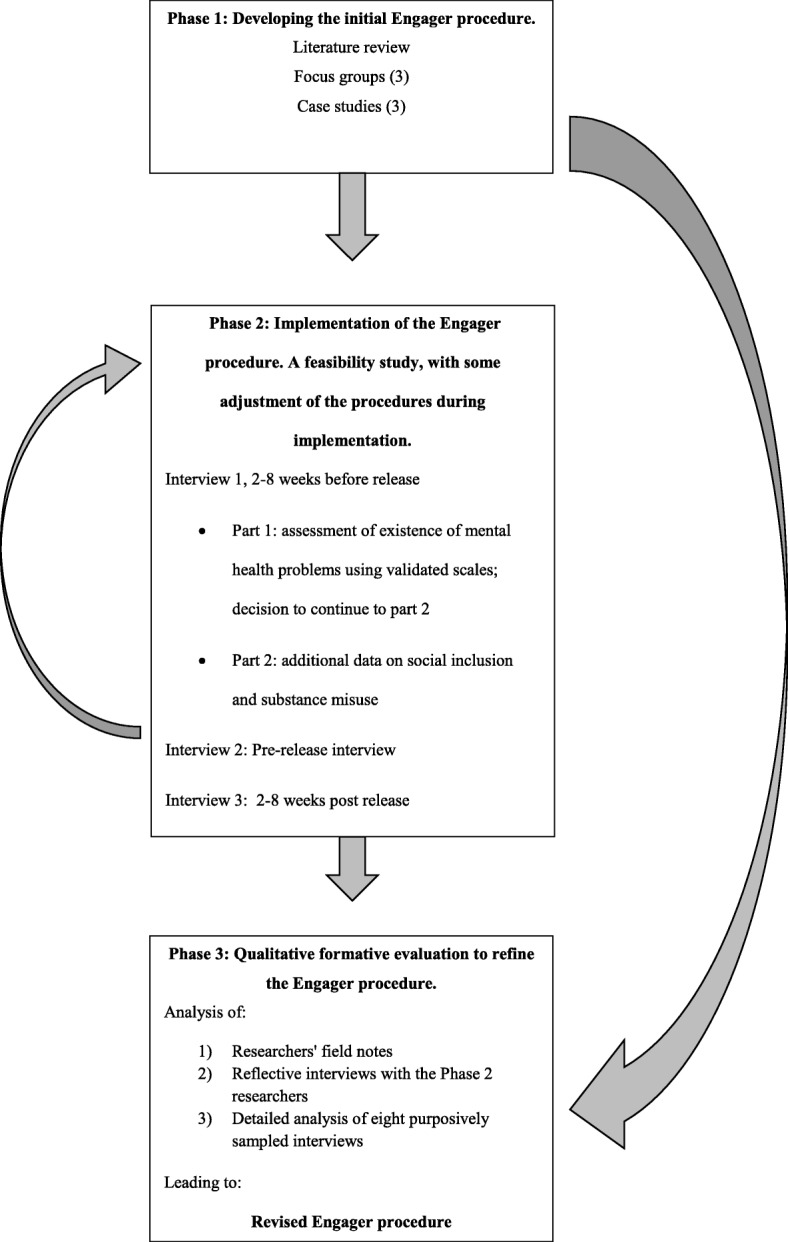
Study design for developing and testing the Engager procedure

Our study used a mixed-methods design to develop and evaluate a procedure for recruitment and retention of prison leavers with common mental health problems in research programmes [17]. The overall design and development of the model is depicted in Fig. 1 . We developed the procedure by carrying out a literature review, case studies, and focus groups (Phase 1). To determine the recruitment and retention rates which could be achieved with the procedure, we trialled it in five prison settings (Phase 2). A formative evaluation was used to further improve the procedure [18] (Phase 3). Ethical approval was obtained from the Research Ethics Committee for Wales (10/MRE09/11) and the National Offender Management Service (Ref no. 22/10).

### Phase 1: development of initial theoretical model and operational procedures for engagement and retention

In order to develop a robust theoretical model and incorporate procedures with high prior probability of utility, we combined evidence from three methods:

- A focussed literature review which identified mechanisms used to engage offenders in research (the search included papers published between 01/2000 and 03/2010 for which full articles in English were available).
- Three focus groups comprising people with experience of being subject to the Criminal Justice System, including prisoners aged 18–21 years, volunteers and users of a ‘through-the-prison-gate’ mentoring service, and members of a support group for families of people in prison. The discussions were audio recorded and transcribed verbatim.
- Case studies of three criminal justice schemes; we undertook documentary analysis and telephone discussions with key staff. The three schemes included a through-the-prison-gate mentoring service, a large-scale longitudinal interview survey of prisoners before and after release, and findings from a research project about informed consent in people with learning disabilities.

A realist informed approach was adopted, and the three data sets were examined for mechanisms that could act as barriers and facilitators to engagement and retention for prisoners in a mental health randomised controlled trial [19]. A ‘mechanism’ was defined by the research team as ‘a phenomenon (changes to systems, and practitioner or researcher behaviours) that could be identified, through its presence or absence, as encouraging a participant to continue to participate or not to remain in the study’. The findings were used to create an initial theoretical model and operational procedures for the collection of data at several stages: initial contact, initial interview, pre-release motivational contact, post-release contact, and 1:2 post-release follow-up interviews (see Fig. 1, Phase 2).

### Phase 2: measuring recruitment and retention using the Engager procedure

#### Participants

We invited male prisoners from five prisons located in two research sites in the North West and South West of the UK to participate in this part of the project, which was designed to mimic the control arm of a randomised controlled trial. The prisons included local prisons with remand capacity (category B) and more settled training prisons (category C). Potential participants were identified through the prisoner record database and approached sequentially if they were serving sentences of less than 2 years, if they were to be released to a defined geographical area, and if they were within 2–8 weeks of their anticipated release date. The option to decline participation was repeated at all stages of the consent process.

Potential participants who consented undertook Part 1 of Interview 1. Those who were not currently receiving treatment for a severe mental illness but who would potentially be suitable to receive an intervention for common mental health problems were given the option to proceed to Part 2 of Interview 1, which was usually carried out as part of the same interview session. Three groups were selected for inclusion in Part 2 based on the presence of current common mental health problems (CCMHP) or past common mental health problems (PCMHP) as follows:

1. Participants with CCMHP: those who scored above the thresholds on the nine-item Patient Health Questionnaire (PHQ-9), seven-item Generalized Anxiety Disorder (GAD-7), and post-traumatic stress disorder (PTSD) scales (scores > 10, > 8, and > 3, respectively)
2. Participants with a likely personality disorder but no CCMHP or PCMHP: those with a positive score on the Standardized Assessment of Personality - Abbreviated Scale (SAPAS) (> 3) but below the cut-offs for the PHQ-9, GAD-7, and PTSD scales
3. Participants not currently ‘positive’ according to the PHQ-9, GAD-7, and PTSD scales but reporting themselves as having had common mental health problems (PCMHP) in the past 2 years: ‘bad stress’, ‘anxiety’, ‘depression’, ‘PTSD’, ‘obsessive-compulsive disorder’, ‘panic attacks’, ‘self-harm’, or an ‘eating disorder’ that had prevented them from normal functioning, or that they thought would do so on release

Participants also had to agree that they would be willing to accept help with the issues that had been discussed in the interview (these were not necessarily framed in diagnostic language) and agree to attend a research interview in the community after release.

#### Measures

In Part 1 of Interview 1, standardised diagnostic tools to measure mental health symptoms, and other quantitative socioeconomic assessments, were embedded in a discursive narrative format in which the researchers discussed the issues that were important to participants in their lives. The diagnostic measures were used as a screening tool to identify individuals who would be considered suitable for a trial for a common mental health intervention. These measures were the PHQ-9 for depression [20], the GAD-7 for anxiety [21], the PTSD screening scale [22], and the SAPAS for personality disorder [23]. In Part 2 of Interview 1, the Michigan Alcoholism Screening Test (MAST) [24], Michigan Drug Abuse Screening Test (DAST) [25], and a social inclusion scale [26] were used to provide further descriptors. All of these measures were repeated at the post-release follow-up interview (Interview 3).

#### Sample size and data analysis

**Fig. 2:**
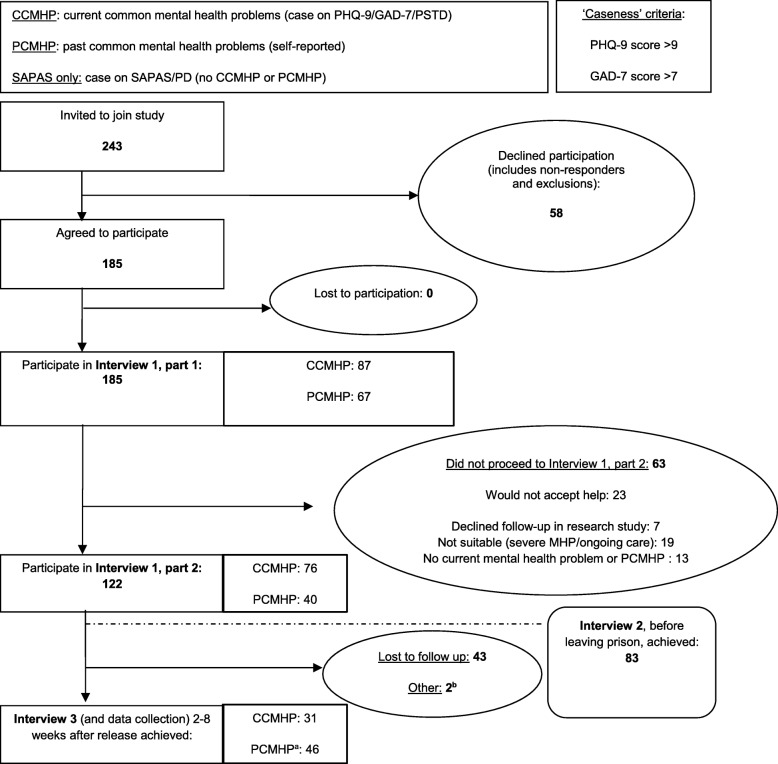
Summary of study participant flow in study and reasons for non-continuation in the study

Based on likely estimates of follow-up in this population [15], we aimed to recruit 100 prisoners, split equally between the two sites, in order to estimate a level of follow-up rate of 60% ± 10% with 95% certainty. The flow of participants has been summarised in Fig. 2. We defined ‘follow-up’ as the number of released prisoners for whom outcome data was collected 2–8 weeks after release as a proportion of all of those identified as having common mental health problems and agreeing to continue to be part of the study on release. Univariable logistic regression was used to assess the association between baseline mental health scores (PHQ-9, GAD-7, PTSD, and social inclusion) and whether a prisoner had outcome data collected.

### Phase 3: qualitative formative evaluation

To optimise the ‘Engager procedure’, which consisted of recruiting and retaining prison leavers who would be suitable to receive an intervention for common mental health problems within a trial format, and to further identify and understand the mechanisms by which it worked, we examined:

1. Issues during the iterative implementation of the Engager procedure, captured through researcher field notes and regular supervision sessions incorporating researcher reflective practice discussions. Some refinements to the Engager procedure were made during implementation of the initial procedure following reflective supervision sessions.
2. Audio recordings of post-implementation reflective interviews with Phase 2 field researchers (DS and SD), who were both asked to reflect on reasons for their actions, which they may not have been consciously aware of at the time, by a qualitative researcher (CQ).
3. Eight, digitally audio-recorded and transcribed verbatim, interviews which were purposively selected to include a range of researchers, sites, participants who had been retained in the study, and those who had been lost to follow-up.

Two qualitative researchers (CS and KD) reviewed the above data, in comparison to the original procedure produced in Phase 1, to test the Phase 1 mechanisms and identify further mechanisms which promoted engagement and retention within the study. The study team then reviewed the Engager procedure, considered the findings of this analysis, and refined the Engager procedure to incorporate the refinements and additional mechanisms indicated by this reflective analysis. Finally, we mapped components of the procedure against the behaviour change ‘functions’ described in an integrated framework of behaviour change interventions, the Behaviour Change Wheel [5], in order to identify how our procedure related to a theoretical behaviour change model.

## Results

We developed a flexible procedure which was successful in recruiting 76% of those invited to participate from a vulnerable population, with challenging life circumstances and competing priorities, to participate in a pilot trial. Engagement with and retention within the trial were also achieved, with 63% of those who met the inclusion criteria for the study attending a follow-up research interview in the community 2–8 weeks after release from prison. Mental health outcomes data was successfully collected at baseline and at follow-up interviews; this process was facilitated by the development of techniques to overcome the high levels of distrust in this population and the stigma associated with mental health diagnoses. We have produced a resource containing the key operational elements of the engagement and retention procedure which could be of use for others developing recruitment and follow-up procedures for other populations who are harder to engage and retain in clinical trials.

### The initial recruitment and retention procedure (Phase 1)

In Phase 1 we developed the initial Engager procedure. The researchers undertook a flexible approach when contacting, interviewing, and following up (potential) participants, particularly when considering where and when people wanted to talk to them. For a group of people who frequently have little control over significant aspects of their lives, such as housing and finance, it was particularly important to demonstrate this degree of respect for their preferences. Trust was facilitated by researchers distancing themselves from association with the Criminal Justice System. This included wearing clothes that distinguished them from custodial staff and using titles or, with participants’ permission, first names; custodial staff generally referred to prisoners by their surnames. The reasons for collecting personal information and contact details were also explained. A recognisable project ‘brand’ logo was developed to further distance the project from the Criminal Justice System and to build on previous, and one hopes positive, interactions. The researchers avoided using potentially stigmatising psychiatric language and diagnostic labels, talking instead about ‘feeling low’, ‘feeling anxious’, or having difficulty coping. Respect was demonstrated for (potential) participants by listening to and validating the issues and concerns which they prioritised. A degree of incentive was used in offering a warm drink and biscuits during the interviews; this was particularly appreciated in the category B prisons where these ‘luxuries’ were harder to obtain and by those who were homeless at the time of the post-release interview. With the participants’ agreement, family members and community services that they were in contact with were included in follow-up plans. These people and services were contacted regularly to re-establish contact with participants whose post-release contact details proved to be insufficient. Contact based on ‘motivational interviewing’ principles, such as ‘rolling with resistance’ and identifying motivators, was made prior to and soon after release to help develop trust and continuity, to understand key motivators, and to proactively problem-solve disruption in contact issues caused by changes in offenders’ circumstances [27, 28].

### Recruitment and retention rates (Phase 2)

In Phase 2 we delivered the Engager procedure derived from the Phase 1 analysis; 185 (76%, 95% confidence interval (CI) 70–81%) of the 243 prisoners invited to participate agreed to take part. Table 1 summarises information about the participants’ criminal justice and sociodemographic status.

**Table 1:** Participant descriptors (*N* = 185)

Descriptor	Mean (standard deviation)
Age (years)^a^	32.70 (10.21)
Number previous prison sentences	7 (11)
Number previous community sentences	3 (3)
Current sentence (months)	9 (7)
	Before prison (*N* (%))
Accommodation^b^	
Significant need	98 (53)
No significant need	65 (35)
Information missing	22 (12)
Employment	
Paid SE/FT/PT	55 (30)
Retired	2 (1)
FT education	4 (2)
Unemployed, looking for work, cannot work	109 (59)
Other	14 (8)

*FT* full-time, *PT* part-time, *SE* self-employed

^a^*N* = 184 for age, as one record had this recorded as ‘OK’

^b^Significant need = residential or sheltered housing, hostel, homeless, living on street, staying with friend or family but with own room, ‘sofa surfing’; no significant need = house or flat owned by participant (including with mortgage), house or flat rented from housing association or local authority, or house, flat, or room rented from private landlord; other = other or missing data

Of these 185 participants, 122 met the inclusion criteria and agreed to be followed up in a research study and so progressed to participating in Interview 1, Part 2. The pre-release interview (Interview 2) was considered to be desirable, but it was not always logistically feasible; 83 of the 122 took part in Interview 2. Of the 122, 77 participants attended a follow-up meeting in the community, Interview 3, in which research data was collected approximately 4 weeks (range 2–8 weeks) after release from prison. This represented a 63% (95% CI 54–71%) follow-up rate of the 122 participants who met the both study inclusion criteria and agreed to take part and be followed up in a research study. Figure 2 shows these results in the form of a flow diagram and details the reasons that potential participants did not continue in the study.

Follow-up interviews were carried out, after prison release, in a location of the participants’ choice. These interview locations included 31 (40%) cafés, 15 (20%) substance misuse services, 11 (14%) probation offices, 2 (3%) in the participants’ General Practitioner’s surgery, and 11 (14%) in prison for participants who had returned to prison and so were interviewed there. No location was recorded for seven (9%) participants. Factors which predicted an increased likelihood of follow-up were reporting themselves to have had common mental health problems in the past (odds ratio = 2.63, *p* = 0.05, CI 0.99–6.96) and showing higher levels of social inclusion on the social inclusion scale [26] (odds ratio = 2.31, *p* < 0.01, CI 1.39–3.86) in Interview 1, indicating that those who experienced higher levels of social inclusion were more likely to be followed up.

### The refined Engager procedure (Phase 3)

In Phase 3 the Engager procedure was refined based on the results of a qualitative formative analysis which considered what had been learnt from implementing the procedure in Phase 2. Additional file 1 (A practical resource for developing recruitment and retention procedures for harder-to-engage populations) details the stages of the procedure, including both the initial components and the refinements made in Phase 3. Additional file 1 can be used as a practical resource for those developing engagement and follow-up procedures for vulnerable and marginalised populations.

Changes to the initial procedure were mainly minor and often subtle refinements. The initial procedure incorporated components designed to overcome key barriers to engagement such as distrust, poor literacy, cognitive deficits, impulsivity, and a resistance to a mental health diagnosis. Our understanding of these issues deepened, and practical ways to address them were developed. Approaching prisoners at their cell door was found to be more effective than sending written invitations. The delicate decision about the researcher’s proximity to the cell door, however, had to be made on an individual basis, balancing the need for confidentiality and avoidance of stigma with respect for the individual’s personal space and the researcher’s personal safety. When using validated scales, if the participant had already spontaneously answered these questions earlier in the interview, the specific question was not repeated. Repetition was avoided because it could make participants feel as if the researcher was not really listening to them; however, this does raise concerns about the reliability of the scoring instruments used. Validated scales are often given to participants to self-complete, which avoids this problem, but this was not considered viable with this population because of the high levels of reading difficulties which prisoners are often reluctant to disclose. Other refinements included being sensitive to participants’ preferences during follow-up — for example, whether they preferred texts or phone calls, the time of day at which they functioned best, and whether they would prefer to meet in a ‘smarter’ café as a ‘treat’ or somewhere more familiar.

Some areas of the Engager procedure were identified as needing further development. For example, the use of a formalised motivational interview prior to release was perceived by researchers to be unsuccessful; this was partly due to time and logistical restraints and also due to a mismatch between participants’ focus on their immediate needs following release and ‘motivating’ someone to attend a research interview which has little immediate or apparent personal benefit. The researchers did make productive use of individual motivational interviewing techniques in the less formal interactions at all stages of the procedure such as ‘rolling with resistance’, involving participants in problem-solving in advance, and identifying their priorities. These could be further developed within the procedure. Other issues identified as not being fully addressed included the prison staff’s variable levels of motivation to facilitate research and the researchers’ degree of familiarity, and hence of comfort and functioning, in different prisons. Awareness of different geographical areas — rural and urban — was also crucial in organising and arranging follow-up, as well as calculating different allowances for travel, which were a significant cost in achieving follow-up interviews. Finally, the analysis suggested that offenders could be proactively involved by including them in the problem-solving about the research challenge of achieving follow-up interviews with themselves.

We explored the degree to which the refined Engager procedure could be specified by the intervention functions identified in the Behaviour Change Wheel [5]. We concluded that the main intervention functions used were the following:

- Researchers used e*ducation* to inform participants of the rationale of the project in order to increase trust; conversely, researchers encouraged prisoners to *educate them* on the best ways to follow them up.
- Researchers used *persuasion* by associating the research process with positive emotions; for example by showing the prisoners respect, linking the research to prisoners’ concerns (e.g. their relationships rather than mental illness), and working collaboratively with the prisoners.
- *Enablement* was important; for example giving individuals confidence by reassuring them that their responses were useful before focussing on key information.

Modelling, environmental restructuring, training, and incentivisation were also used to a lesser degree. *Modelling* was used in a limited way, although not at the instigation of the researchers. Some participants chose to attend the initial interview because other prisoners recommended it as a positive experience. Whether the chance to have a break from their cell, to join a discussion with a new person, and have a hot drink and biscuits can be considered *environmental restructuring* is questionable, but the ambiance and acceptability of the location for community-based interviews was of great importance for some participants. By encouraging participants to consider how they could be contacted in the community and encouraging them to participate in problem-solving, researchers provided a limited amount of *training*. The offer of a hot drink and biscuits, the award of certificates for participation, and the gratuity of a voucher for attending community interviews (to thank people for their time and contribution) could all be considered forms of *incentivisation. Restriction* and *coercion* were not direct, although it could be argued that some prisoners felt they had to comply; researchers actively worked against this by making it very clear at the consent stage that prisoners could return to their cell and the prison staff would be told that they had done everything they had been asked.

## Discussion

This study illustrates that it is possible to engage and retain offenders with common mental health problems in research. Retention rates of 38–99% have been achieved with prisoners receiving opiate substitution and/or counselling for substance misuse, treatment for HIV, mental health intervention, or help for nursing mothers [9, 10, 12–16]; these results were obtained in a variety of types of study design. We were able to recruit 76% of those invited, and 63% of those who met the inclusion criteria and were willing to participate in a research study attended a follow-up interview 4–8 weeks after release from prison. Our searches located no directly comparable studies mimicking the control arm of a randomised controlled trial for a prison release population. While the 63% figure falls short of the 80–87% retained in the best trials of mental health interventions, these higher values are obtained in populations without the levels of distrust and chaos of prison leavers [29–31]. The 63% figure was also achieved in the absence of even the possibility of being randomised to an attractive intervention. Although this may possibly have reduced recruitment rates, it may have increased retention rates as there was no potential disappointment from not being randomised to an intervention. The revised procedure has been successfully used in the subsequent Engager 2 two-arm pilot and randomised controlled trials [32, 33]. The pilot trial achieved a follow-up rate of (73%, 95% CI 61–83%) at 1 month post release and (47%, 95% CI 39–59%) at 8–15 weeks post release. The early findings for the main trial have demonstrated a follow-up rate of 184/277 (66.4%) at approximately 6 months post release.

We developed a range of practical and potentially important procedures both for engaging an initially authority-distrustful population in research and for achieving follow-up when this population was no longer subject to a punitive authority. These procedures included developing an interactive relationship with the participant, using bespoke follow-up procedures for individuals, gaining alternative contacts details, developing a project brand, and using rewards. Previous work on participant recruitment and retention in mental health trials has revealed a variety of ways by which follow-up rates can be increased, some of which were also identified as helpful in our own work. For example, Ribisl et al. found that participant attrition could be minimised by creating a project identity, making research involvement convenient and rewarding for the participant, and customising research processes to individual studies and participants [34]. More recently, Bell et al. and Arean et al. identified monetary incentives and having the same member of the research team for each contact, which builds rapport and trust, as important for increasing participant retention [35, 36]. Bell et al. also found that ‘branding’ a research project, having frequent contact, and tailoring the timing of contact to the preference of the participant increased retention [35]. Both Barrowclough et al. and Bell et al. noted that persistence and flexibility are important — for example, making repeat visits for missed appointments and allowing participants to miss single visits without having to drop out of trials completely [35, 37]. Although motivational interviewing was not implemented as originally intended, some of its techniques were perceived as a useful tool when used flexibly by researchers, in line with the finding of McMurran et al. that motivational interviewing can increase retention [38].

### Strengths and limitations

This study used a realist informed mixed-methods approach to both develop and refine the ‘Engager procedure’. This approach allowed mechanisms from a range of contexts and multiple perspectives to be incorporated into the final procedure. The multi-method, primarily qualitative approaches only allow us to present hypotheses about which components are most critical.

### Implications for research

This study has shown that it is possible to gain adequate retention rates for the control arm of a trial of British male prison leavers with common mental health problems. In line with the conclusions of Michie et al. [5], we emphasise the importance of understanding prisoners’ thinking and behaviour in context in order to develop a clear method for engaging and retaining them in research. These results have been proven to be applicable to other prison leaver settings and may be applicable to a wider range of hard-to-reach groups, although it would be important to apply them within a detailed understanding of each context.

In order to implement research protocols such as this, it is important to be specific about the protocol and how to implement it, but also to be aware that the protocol is unlikely to capture all the tailoring necessary, given the personal and dynamic nature of interactions upon which the success of implementation depends. Protocols need to emphasise both the range of approaches for achieving a flexible, personalised follow-up procedure and also that it may be necessary to respond flexibly in ways not detailed in the procedure while still respecting ethical concerns such as fully informed consent.

### Implications for practice

Many of the strategies that have been identified here could also be used by clinicians trying to engage vulnerable and socially marginalised groups, for example, developing trust by using non-stigmatising language, using flexible practices which respond to the individual’s social situation, and presenting healthcare as separate from the prison system.

## Conclusions

It is possible to engage and retain offenders with common mental health problems in research requiring collection of mental health outcomes, and our retention rate of 63% is comparable with the best of other trials of prisoners with different health conditions after release internationally [39, 40], with the exception of substance misuse trials.

The strategies identified, detailed in Additional file 1, require some flexibility to deviate from standardised trial protocols as well as personable and tenacious researchers, but they are not expensive to implement. Many of the procedures are potentially transferable to the clinical tasks of engaging with and following up individuals who have high levels of distrust of systems and authority and with whom it is difficult to maintain contact through conventional means.

## Additional file

Additional file 1: A practical resource for developing recruitment and retention procedures for harder to engage populations. (DOCX 26 kb)

## Abbreviations

CCMHP: Current common mental health problems
DAST: Michigan Drug Abuse Screening Test
GAD-7: Generalized Anxiety Disorder (scale)
MAST: Michigan Alcoholism Screening Test
PCMHP: Past common mental health problems
PHQ-9: Patient Health Questionnaire
PTSD: Post-traumatic stress disorder
SAPAS: Standardised Assessment of Personality - Abbreviated Scale
