# Supplementary material for: The feasibility of following up prisoners, with mental health problems, after release: a pilot trial employing an innovative system, for engagement and retention in research, with a harder-to-engage population
Source: Trials. 2018 Oct 1;19:530. doi: 10.1186/s13063-018-2911-1 (PMC6167907; doi:10.1186/s13063-018-2911-1)
Supplement: Supplementary file 1 — A practical resource for developing recruitment and retention procedures for harder to engage populations. (DOCX 26 kb) [file 13063_2018_2911_MOESM1_ESM.docx]

**Additional file 1: A practical resource for developing recruitment and retention procedures for harder to engage populations. ***

| **Study Stage:** | **Recruitment:**   - Approach identified potential participants on the prison wing. - Talk about the study and invite potential participants to discuss involvement in more detail. |
| --- | --- |
| **Procedures:** | **Rationale:** |
| Describe the study in general non-stigmatising terms. | This helps to overcome the stigma associated with mental health diagnostic labels and can also help to avoid bullying, particularly concerning mental health, as others may overhear what you are saying. |
| Emphasise participants’ priorities, such as resettlement issues, rather than mental health. | This demonstrates that you are interested in their concerns and recognises that mental health concerns are unlikely to be their highest priority. |
| Use friendly face-to-face approaches; e.g. at the cell door, rather than sending information and appointments in the internal post. | Individual contact, where you reach out to them, helps to build trust. |
| Use enthusiastic approaches. | This helps to communicate that you think that they are deserving of your time and attention and that you may have something useful to offer. |
| Work flexibly to fit in with both the prison systems and participant preferences. | This demonstrates that you have recognised that their needs and priorities are also important. |
| Foster respect. Do not automatically enter potential participants’ cells. Use the name Mr X initially, and only progress to first names if invited to do so (custodial staff routinely refer to prisoners by just their surnames). | This demonstrates that you think they are worthy of this respect which helps to overcome distrust. |
| Demonstrate respect for custodial staff. Develop an understanding of the limits and pressures within which they are working. | Custodial staff are often working within strict limits and under numerous pressures. The majority have the prisoners, and your, best interests at heart. |
| **Study Stage:** | **Consent:**  Discuss in a private room where potential participants and researchers cannot easily be overheard. |
| **Procedures:** | **Rationale:** |
| Make sure that potential participants understand what study participation involves and that there are no penalties for not taking part. Techniques to achieve this include the use of easy-to-read sheets and reading out consent forms and information sheets. Potential participants may have reading difficulties that they don’t like to admit to, or have attention drawn to. | Ethically, it is important to go beyond what more conventional ethical procedures might require, to ensure that potential participants have understood what they are agreeing to, or that they can choose not to agree to take part. |
| Clearly explain the purpose of signing the consent form, that the interview will be recorded, and how the information that participants give will be used. | This population can have higher than usual levels of concern about these processes; for some participants the last time they signed a form and had an interview recorded may have been in a police station. |
| Empower through information. Clearly explain the process of being involved, include reassurances that there will be no nasty surprises, that they can take breaks, and that they do not have to answer all of the questions. | This gives potential participants a sense of control which is often absent in their current situation. |
| Use branding; make repeated use of the study name and logo. | This reinforcement and subsequent recognition can help to build ‘brand’ loyalty and make participants feel part of something larger. |
| **Study Stage:** | **Interview 1 (in prison):**   - For those who have consented to participate, conduct interview (Interview 1, part 1) to screen for current common mental health problems (CMHPs), self-reported past common mental health problems (PCMHPs) which have affected participants’ ability to function in the past two years, and those who feel they will have problems coping on release. - Conduct interview (Interview 1, part 2) for those meeting the criteria for any of the above and who are willing to accept help on release and continue to participate in the study. |
| **Procedures:** | **Rationale:** |
| Establish a friendly atmosphere. Use a conversational tone and provide a hot drink and biscuit. | This can help make the participant feel more comfortable talking to you. |
| Pace the interview and do not rigidly enforce the structure of the interview schedule. Start with easy-to-answer, open questions. Maintain a conversational tone. | Starting gently allows participants to build their confidence and feel that they have something worthwhile to say; more nervous participants respond particularly well to this approach. Maintaining a conversational tone helps to mitigate the ‘lists’ of standardised schedule; participants are used to responding to tick-list schedules which do not have any positive impact on the care that they receive. |
| Adopt a holistic approach; allow participants to discuss their wider stories and anecdotes. | Discussing what participants are more interested in helps them to feel more comfortable and listened to, which promotes ongoing engagement. |
| Use an individualised approach, focus on individual preferences to completing the process, and involve participants in these decisions; e.g. some participants respond positively to verbal tick lists while others may prefer a more interactive approach and pointing at laminated response cards. | This approach reinforces that their preferences are important. Adopting individuals’ preferred approaches also helps to overcome barriers to engagement, such as learning disabilities. |
| Demonstrate listening, reflect back information that has been given earlier in the interview process, particularly in the form of ‘life story’ or ‘anecdotal’ accounts outside the conventional format of interview schedules. Acknowledge when a subsequent question asks for repetition of information that has already been given, by verbally recognising that you do not require participants to repeat themselves. | Validating that you have listened to, and retained, what participants have already told you, despite the requirements of the interview schedule, reinforces that you are listening to them as a ‘whole person’, which helps to build trust and ongoing engagement. |
| Explain difficult questions and check participants’ understanding in a supportive way. | Working with participants in this way validates that you believe that participants have something valuable to contribute, even if the question appears to be complicated, and reinforces that all participants can make valuable contributions. This contributes towards building trust. |
| Use sensitivity when asking questions that might cause participants distress, for example, questions regarding suicidal intent. Dropping your voice and making direct eye contact may support this. | Some questions may be required to collect mental health outcomes data, but may also be troublesome for participants who regard mental health diagnostic labels as stigmatising. Employing these techniques may help to overcome sensitivities, and potential stigmatisation, barriers. |
| Use positive encouragement, such as praising achievements, and give certificates of participation. | Reinforcing what participants have done well can contribute to their belief that they can continue to achieve these, and other, positive goals. |
| Respect participants’ boundaries, e.g. do not challenge what participants say about their drug use, or if they claim that they do not to have contact information for people who you might want to try and follow them up through. | Demonstrating respect can help to build trust; this is more important than pushing to gain information that you are unlikely to be given. |
| Encourage participants to proactively contribute to achieving the research aims, e.g. considering the best ways for you to contact them for the follow-up interview. | Suggestions for follow-up methods from participants are more likely to be effective for that individual. Collaboratively involving participants in the research process can promote ongoing engagement. |
| Be aware of the language that you use, allow participants to speak naturally for them, understand their use vernacular terms, use terms such as ‘feeling low’ and ‘feeling stressed’ rather than medicalised mental health ones. | Allowing participants to speak in a way, and using terms, that are familiar to them will help them to feel more comfortable, and therefore more confident, in communicating with you; this can contribute to building trust. |
| Be aware of other pressures on participants, e.g. operational pressures such as returning in time for lunch and other influences such as fatigue. | If parts of the interview process that are more critical than others for achieving engagement and retention routinely conflict with other pressures on participants, the study is less likely to be successful. Our study moved the collecting of follow-up details, which is probably the most important component of retention, from the end to the middle of this interview to avoid potential conflicts with these other concerns. |
| Record as many follow-up details and methods as possible, including mobile phone numbers, family phone numbers (particularly participants’ mothers’ phone numbers, when appropriate) and services that participants have agreed you can contact them through. | Participants routinely change mobile phone numbers and their lifestyles are likely to be unsettled immediately after release from prison. Some participants will not know where they will be living after release from prison when this interview is undertaken. |
| **Study Stage:** | **Interview 2 (before leaving prison):**   - Continue to develop the relationship. - Use motivational interviewing techniques, as appropriate. - Renew follow-up contact information. |
| **Procedures:** | **Rationale:** |
| Continue to build a relationship with the participant, demonstrating that you have remembered what they told you last time by checking your notes in advance and reflecting some of this back to them. | Seeing someone from the study for a second time helps to build a relationship between the researcher and participant and loyalty to the study. If this meeting is a pleasant experience for them, participants are more likely to attend the research interview after prison release. Repeating back to participants some of what they told you in the first interview demonstrates that you were listening and that what they have to say is valuable to you and the study. |
| Use a personable conversational format for this interview; there are no tick-boxes to fill in. | The lack of a generic pre-set interview schedule makes it easier to talk about participants’ lives more holistically at a time when they are likely to be anxious about release related issues and may find it difficult to take part in something more demanding which does not address their immediate concerns. |
| Use motivational interviewing techniques, as appropriate, to encourage attendance at Interview 3. The techniques used could include: reflecting back to people; being non-judgemental by not challenging resistance; validating the positive; being solution focused for resettlement issues; and being solution focused on the most likely ways for follow-up to occur. | Involving participants in thinking about what might stop them, personally, attending Interview 3 and working in partnership with them, using their knowledge of their own circumstances, reinforces that their contributions to the process are valuable and should make it more likely that they will attend Interview 3. |
| Review, and if possible add to, follow-up contact details. | These details, and their resettlement plans, may have changed since you last saw them. |
| **Study Stage:** | **Interview in the Community:**   - Takes place 2-8 weeks after release from prison. - Conduct interview 3; main outcome measure for the study. - When relevant review and renew contact details for 12 week post-release interview. |
| **Procedures:** | **Rationale:** |
| Try to make early contact with participants. The process of following participants up should start at two weeks after their release, rather than four. | It could take an additional two weeks to find, establish contact and arrange a meeting with someone. This procedure effectively replaced the immediate post-contact release that we had originally planned, except for those participants who had specifically requested this. |
| Work flexibly around participants’ needs regarding the time and location of the interview. Potential locations could include places they are attending for other appointments (addiction services and probation) and places that have no association with the Criminal Justice System, such as cafés. Allow participants to choose a location which meets their priorities such as a nicer café, which feel like a treat, or local café, which feels more comfortable. | The more comfortable and convenient it is for participants to attend the interview, the more likely they are to attend. |
| Establish and build up relationships with local organisations used by participants (addiction services, probation and homeless hostels. Invest time in ‘building up one’s patch’; this is more challenging in more geographically dispersed areas. Build positive relationships with the ‘gatekeepers’, often receptionists, at these organisations. | This is essential, particularly for those participants who do not have access to more conventional ways of being contacted, such as a stable address. |
| Be aware that participants’ post-release experiences seem to influence their motivation to attend this interview. When arranging the interview, if they seem reluctant to attend, consider reminding them that you are not judging them or assessing them in this way. | Participants who are doing well seem motivated to share this. Participants who are not doing as well as they had hoped, seem less motivated to take part in this interview, attendance is more likely to be achieved through contact with community services or back in prison. |
| When arranging the interview, if appropriate, remind them that you are interested in, and would like to hear about how things have gone for them since you last saw them. | Although other things in the community were competing for participants’ attention, someone to listen to them remained a strong motivating factor for attendance. Speaking to someone outside Criminal Justice services, who would not judge or punish them, and to a non-family member, who would not be burdened or demonstrably upset by what they said, was particularly valued. |
| Be sensitive to some participants not wishing to attend because they do not want to do anything associated with ‘the service’ after leaving prison. | While researchers can try to address participants’ concerns about attending an interview, there are times when it is more appropriate to step back and respect their preferences. |
| Be proactive and persistent in your follow-up techniques, without harassing people. Recording the range of follow-up approaches that you have tried for each individual will help to balance these, potentially competing, concerns. Continue with this persistence, even if community services say that attendance is unlikely. | This population can be particularly difficult to follow up and are more likely to respond positively to someone demonstrating that they have reached out to them, rather than just being required to attend another appointment. Researchers have a different relationship with participants, than community services do, and they are offering something different. |
| Consider the use of a range of contact approaches which take into account an individual participant’s needs, e.g. some participants may not be able to read texts easily, some may find phone calls more intrusive and others may dislike being contacted in the morning/afternoon. | Being aware of, and respecting, individuals’ needs means that they are more likely to respond positively to your request for an interview. |
| Develop and maintain the relationship. This can be done by referring to things that participants have previously told you during conversations to set up interview and in interview itself; use your researcher notes from previous interviews to support this. | This helps participants feel that they, and what they have to say, are valued. |
| Be conscious of the value of gratuities/incentives. A mobile phone top up voucher was appreciated and gratefully received. | This acknowledges that participants’ time and contribution is valuable; this procedure is routine in most, main population, healthcare trials. |
| Consider safety issues; these can include the careful use of research buddies (who attend the interview, are introduced to the participant and then sit where they can see, but not hear, the interview taking place) and rigorous lone worker procedures. | No community-based, in person, research is without risks and these should be evaluated on a per participant, and per location, basis to support researchers in carrying out their role. |
| **Study Stage:** | **12 weeks, post-prison release, interview.**   - Retain engagement and repeat Interview 3. - Carried out, when possible, to gain additional learning for the study. |
| **Procedure:** | **Rationale:** |
| Regularly repeat the engagement techniques used for Interview 3. | To maintain the relationship and also because contact details can change quickly, even at this stage of the post-prison release period. |

*At the end of Phase 3 of the research process.
